# Sperm DNA Hypomethylation Proximal to Reproduction Pathway Genes in Maturing Elite Norwegian Red Bulls

**DOI:** 10.3389/fgene.2020.00922

**Published:** 2020-08-11

**Authors:** Abdolrahman Khezri, Birgitte Narud, Else-Berit Stenseth, Teklu Tewoldebrhan Zeremichael, Frøydis Deinboll Myromslien, Robert C. Wilson, Rafi Ahmad, Elisabeth Kommisrud

**Affiliations:** Department of Biotechnology, Inland Norway University of Applied Sciences, Hamar, Norway

**Keywords:** Norwegian Red, bulls, puberty, sperm, DNA methylation, RRBS

## Abstract

Genomic selection in modern farming demands sufficient semen production in young bulls. Factors affecting semen quality and production capacity in young bulls are not well understood; DNA methylation, a complicated phenomenon in sperm cells, is one such factors. In this study, fresh and frozen-thawed semen samples from the same Norwegian Red (NR) bulls at both 14 and 17 months of age were examined for sperm chromatin integrity parameters, ATP content, viability, and motility. Furthermore, reduced representation bisulfite libraries constructed according to two protocols, the Ovation^®^ RRBS Methyl-Seq System (Ovation method) and a previously optimized gel-free method and were sequenced to study the sperm DNA methylome in frozen-thawed semen samples. Sperm quality analyses indicated that sperm concentration, total motility and progressivity in fresh semen from 17 months old NR bulls were significantly higher compared to individuals at 14 months of age. The percentage of DNA fragmented sperm cells significantly decreased in both fresh and frozen-thawed semen samples in bulls with increasing age. Libraries from the Ovation method exhibited a greater percentage of read loss and shorter read size following trimming. Downstream analyses for reads obtained from the gel-free method revealed similar global sperm DNA methylation but differentially methylated regions (DMRs) between 14- and 17 months old NR bulls. The majority of identified DMRs were hypomethylated in 14 months old bulls. Most of the identified DMRs (69%) exhibited a less than 10% methylation difference while only 1.5% of DMRs exceeded a 25% methylation difference. Pathway analysis showed that genes annotated with DMRs having low methylation differences (less than 10%) and DMRs having between 10 and 25% methylation differences, could be associated with important hormonal signaling and sperm function relevant pathways, respectively. The current research shows that RRBS in parallel with routine sperm quality analyses could be informative in reproductive capacity of young NR bulls. Although global sperm DNA methylation levels in 14 and 17 months old NR bulls were similar, regions with low and varying levels of DNA methylation differences can be identified and linked with important sperm function and hormonal pathways.

## Introduction

Epigenetics is a phenomenon where gene expression is regulated without any changes in DNA sequence, rather being modulated via changes in DNA methylation, histone post-translational modification, and interaction of transcriptional factors with small RNAs (Donkin and Barres, 2018). Epigenetic changes in sperm cells are even more complex compared to somatic cells for two main reasons. First, during primary phase of spermatogenesis, where germ cells develop to spermatids, DNA methylation is initially erased, becoming re-established later. Moreover, during spermiogenesis, where spermatids further differentiate to spermatozoa, the majority of histones are gradually replaced by protamines (O’Doherty and McGettigan, 2015; McSwiggin and O’Doherty, 2018). In recent years, different methods have been developed to study DNA methylation. Reduced representation bisulfite sequencing (RRBS) is an efficient and high-throughput method, allowing the study of DNA methylation profiles at single-base resolution, while experiment costs are kept low (Meissner et al., 2005). Previous studies have used RRBS to investigate DNA methylation profile in different bovine somatic tissues as well as bull sperm cells (Zhou et al., 2016; Perrier et al., 2018).

Sexual maturation in bulls is a hormone-regulated process lasting up to 50 weeks of age (Rawlings et al., 2008). Previous research has demonstrated that semen quality is closely correlated with different environmental factors and animal age. For instance, it has been shown that sperm morphology, concentration and motility positively correlated with age in young tropical composite bulls (Fortes et al., 2012) and Austrian Simmental bulls (Fuerst-Waltl et al., 2006). Although several studies reported low methylation levels in different genomic features of bull sperm cells (Perrier et al., 2018; Zhou et al., 2018; Kiefer and Perrier, 2019), the methylation level is dynamic and recent evidence suggests that the bull sperm methylome correlates with age (Lambert et al., 2018).

Norwegian Red (NR) is a highly fertile breed with e.g., low incidence of calving difficulties and mastitis (Refsdal, 2007; Ferris et al., 2014). Historically the NR breeding program was based on progeny testing of sires. However, starting from 2016, genomic selection was implemented in the NR breeding program and top NR elite bulls are today selected based on genomic breeding values. Despite the fact that NR is widely employed for artificial insemination (AI) in Norway and a very good record of genetic information has been build up during the last 40 years, little is known about the NR sperm methylome. In addition, because of short generation intervals due to genomic selection, there is more demand hence more physiological pressure for semen production from young NR bulls. The objective of this research is to determine if sperm DNA methylome could provide additional information to age related sperm quality differences. For this purpose, sperm samples from the same NR bulls at 14 and 17 months of age were used for analyzing chromatin integrity, viability, ATP content, and motility parameters. Furthermore in order to assess the sperm DNA methylome, two different RRBS protocols including Ovation RRBS Methyl-Seq System (as a, simple, fast, and scalable solution) and a gel-free based RRBS protocol which previously was optimized to study boar sperm DNA methylome (Khezri et al., 2019), were implemented.

## Materials and Methods

In the present study, the sperm quality traits including chromatin integrity parameters, ATP content, viability and motility parameters were analyzed in semen from NR elite bulls, at both 14 and 17 months of age. Furthermore, following comparison of two different protocols for constructing RRBS libraries, sperm DNA methylation in both age groups was analyzed.

### Semen Collection and Sample Preparation

Bulls in this study, were raised, cared for and fed standardized diet at Geno SA (Geno Breeding and AI Association, Hamar, Norway), AI station. Ejaculates were collected from nine young genomic selected NR bulls with unknown fertility at 14 and 17 months of age and processed by the breeding company Geno SA. Prior to dilution, sperm cell concentration in each ejaculate was calculated using an Accucell^®^ spectrophotometer (IMV Technologies, L’Aigle, France). The semen was diluted in a two-step procedure using Biladyl extender (13500/0004-0006; Minitübe, GmbH, Tiefenbach, Germany). After first dilution, samples were taken for fresh semen analyses and simultaneously used for subjective quality control analysis. Ejaculates with motility above 70% and morphological abnormalities below 20% were further diluted with the glycerol-containing fraction (1:2) to a final concentration of 12 × 10^6^ sperm cells per insemination dose, filled into 0.25 ml standard French mini straws (IMV, L’Aigle, France), and cryopreserved as previously described (Standerholen et al., 2014). Cryopreserved doses were later prepared for sperm quality analyses and DNA extraction by thawing the semen doses for 1 min in a water bath at 37°C. To minimize the influence of possible variation between straws, semen from two straws/ejaculate were pooled and mixed before analyses.

### Sperm Quality Analyses

#### Sperm Chromatin Integrity Assessment

Sperm chromatin integrity assessment was performed using sperm chromatin structure assay (SCSA) (Evenson and Jost, 2000; Boe-Hansen et al., 2005). Using this assay, two different chromatin integrity parameters, including DNA fragmentation index (DFI) and high DNA stainability index (HDS), were measured.

In brief, both fresh and frozen-thawed semen samples were diluted to 2 × 10^6^ cells/ml using TNE buffer (10 mM Tris-HCl, 0.1 M NaCl, 1 mM EDTA, pH 7.4). Diluted samples were denatured for 30 s by adding an acid detergent solution (0.38 M NaCl, 80 mM HCl, 0.1% Triton-X 100, pH 1.2). Denatured samples were stained with acridine orange (AO) staining buffer (37 mM citric acid, 0.126 M Na_2_PO_4_, 1.1 mM EDTA, 0.15 M NaCl and 0.6 μg/ml of AO, pH 6.0) and were incubated for 3 min at room temperature. Two technical replicates were considered for each sample and 5000 sperm cells per replicate were analyzed using a pre-AO saturated flow cytometer equipped with a blue laser (488 nm) (Cell Lab QuantaTM SC MPL flow cytometer, Beckman Coulter, Fullerton, CA, United States). Laser stability was controlled (at the beginning of the experiment and after every fifth sample) using a bull reference sample with pre-identified DFI and by re-setting the mean green and red fluorescence signals to 425 ± 5 and 125 ± 5, respectively. Following AO staining, double- and single-stranded DNA emitted green (collected using a 525 nm band pass filter) and red fluorescence (collected using a 670 nm long pass filter), respectively. The percentage of red and green fluorescence was determined using the FCS Express 6 flow cytometry data analyzer software (*Denovo* Software, Los Angeles, CA, United States). Based on the ratio of red/(red + green), the DFI percentage was calculated. Furthermore, HDS sperm cells, which are considered as sperm cells with an incomplete chromatin condensation, were identified according to a high incorporation of AO into double-stranded DNA.

#### Assessment of Sperm Viability

Sperm viability and data analysis were performed using the flow cytometry system described above and Kaluza^®^ software, Version 2.1 (Beckman Coulter Ltd.). Frozen-thawed semen samples were diluted in SP-Talp media (105 mM NaCl, 3.1 mM KCl, 0.4 mM MgCl_2_, 2.0 mM CaCl_2_⋅2H_2_O, 0.3 mM NaH_2_PO_4_⋅H_2_O, 1 mM sodium pyruvate, 21.6 mM sodium lactate, 20 mM Hepes, 20 mM Hepes salt, 5 mM glucose, 50 μg/ml gentamycin) to a concentration of 1 × 10^6^ sperm cells/ml. Two technical replicates were considered per sample. Sperm suspensions were stained with 0.48 μM propidium iodide (PI, Sigma-Aldrich) and incubated for 10 min prior to flow cytometric analysis. PI fluorescence was detected using a 670 nm long pass filter (FL3), and gating was performed to reveal sperm cells population (based on electronic volume) and percentages of living spermatozoa as previous described (Standerholen et al., 2014).

#### Sperm ATP Content

The ATP content was measured in frozen-thawed semen samples using the CellTiter-Glo^®^ Luminescent Cell Viability Assay (Promega; Madison, Wisconsin). A total volume of 60 μl of semen (3 × 10^5^ sperm cells) was added to a white 96-well microtiter plate (NUNC^®^, Denmark) and mixed with 60 μl CellTiter-Glo^®^ reagent. To induce cell lysis, the mixture was gently shaken for 2 min in a rotary shaker (IKA^®^ MS 3 digital, United States), followed by 15 min incubation at room temperature to stabilize the luminescence. The bioluminescence signal was measured in relative luminescence units (RLU) using a FLUOstar OPTIMA multiwell plate reader (BMG LABTECH GmbH, Offenburg, Germany), equipped with MARS data analyzer software (Version 1.10, BMG LABTECH, Germany). Obtained RLU signals were converted to a corresponding ATP value in nM according to a prepared standard curve. ATP values obtained from the average of three technical replicates per sample. Then the value further corrected for the percentage of motile sperm cells before statistical analyses.

#### Sperm Motility Analysis

Sperm motility analysis were performed using the SCA evolution CASA system (Microptic SL, Spain), equipped with a phase contrast Eclipse Ci-S/Ci-L microscope (Nikon, Japan), a BASLER Ace acA780-75 gc digital camera (Basler Vision Technologies, Ahrensburg, Germany) and Sperm class analyzer software (v 6.1.0.0). Fresh and thawed semen samples were incubated for 15 min at 37°C, and diluted (1:2) with pre-warmed PBS buffer (37°C) before analysis. A volume of 3 μl of diluted samples was loaded into the chamber of a pre-warmed (37°C) 20 μm depth Leja-4 slide (Leja products, Nieuw-Vennep, the Netherlands). Analyses were performed using two technical replicates per sample, under a 10x objective and on the pre-heated thermal stage (37°C) of the phase contrast microscope. Eight or more microscope fields with at least 800 cells per sample were analyzed. Bull sperm cells were detected based on head area (20–80 μm^2^) and camera setting of 45 frames per sec. The motility parameters measured were total motility, progressive motility, and hyperactive motility. In addition, other information regarding to sperm motion kinetics including curvilinear velocity (VCL, μm/s), straight-line velocity (VSL, μm/s), average path velocity (VAP, μm/s), straightness of the average path [STR (%) = VSL/VAP], linearity of the curvilinear path [LIN (%) = VSL/VCL], Wobble [WOB (%) = VAP/VCL], lateral displacement of sperm head (ALH, μm) and beat cross frequency (BCF, Hz) were obtained. Sperm cells were defined as static and progressive motile if VAP < 10 μm/s and STR > 70μm/s, respectively. Sperm cells with VCL > 80μm/s, ALH > 6.5μm and LIN < 65% were defined as sperm cells with hyperactive motility.

### RRBS Library Preparation

Prior to RRBS library construction, DNA from frozen-thawed sperm samples of seven bulls at, both 14 and 17 months of age, was extracted using a Maxwell 16 Benchtop instrument (Promega Corporation, United States) at Biobank AS, Hamar. Isolated DNA was quantified using Qubit dsDNA BR assay kit (Thermo Fisher Scientific, United States) and further diluted to 20 ng/μl in low TE media [10 mM Tris, pH 8.0 (Calbiochem, United States), 0.1 mM EDTA, pH 8.0 (Calbiochem, United States)]. Libraries for sperm DNA methylation analysis were constructed using the RRBS approach and according to two different RRBS protocols.

#### RRBS Library Preparation Using Ovation^®^ RRBS Methyl-Seq System (Ovation Method)

In this method Ovation^®^ RRBS Methyl-Seq System (NuGEN Technologies, San Carlos, CA, United States) was employed and RRBS libraries were constructed using 100 ng genomic DNA, according to the manufacturer’s protocol with slight modifications.

Briefly, genomic DNA was digested overnight at 37°C using *Msp*I and Taq α1 enzymes (New England Biolabs, United States). After digestion, AMPure XP beads (Beckman Coulter, United States) were added (2x) and samples were kept at room temperature for 30 min. Then by putting the samples on a side magnet, supernatant was removed and beads were washed twice with 100% EtOH. Dried beads were re-suspended in 10 μl elution buffer (Qiagen, Germany) and fragmented DNA was ligated with adapters by incubation at 25°C for 30 min followed by 70°C for 10 min. Adapter ligated fragments were final repaired at 60 and 70°C each for 10 min. The fragments were further size selected by adding 1.5x of 20% PEG 8000/2.5 M NaCl (Amresco Inc., United States) followed by incubation for 30 min at room temperature. The supernatant was removed as previously described and after washing the beads twice with 70% EtOH and drying, the beads were re-suspended in 25 μl elution buffer (Qiagen, Germany). Eluted products were subjected to bisulfite conversion using EpiTect kit (QIAGEN, Germany) following the manufacturer‘s protocol designated for DNA extracted from FFPE tissues. Bisulfite converted DNA, was amplified using 10 cycles of PCR. Amplified libraries were purified by adding 1x SPRI AMPure XP beads followed by incubation for 30 min at room temperature. Supernatant was removed, beads were washed with 70% EtOH and re-suspended in elution buffer. Eluted beads were further placed on a side magnet and purified libraries were transferred to a clean tube.

#### RRBS Library Preparation Using a Gel-Free Multiplexed Method (Gel-Free Method)

In this method RRBS libraries were constructed using a gel-free multiplexed technique (Boyle et al., 2012), which we previously optimized it to study boar sperm DNA methylome (Khezri et al., 2019). The protocol was consisted of the following steps.

First, genomic DNA (100 ng) was digested as described in section “RRBS Library Preparation Using Ovation^®^ RRBS Methyl-Seq System (Ovation Method).” Gap filling and A-tailing steps were carried out by adding 1 μl of Klenow fragment (New England Biolabs, United States) along with 1 μl of dNTP mixture containing 10 mM dATP, 1 mM dCTP, and 1 mM dGTP (New England Biolabs, United States) to fragmented DNA. The processed DNA was incubated for 20 min at 30°C followed by 20 min at 37°C. After incubation, fragmented DNA was size selected (300–500 bp) by adding a 2x AMPure XP beads (Beckman Coulter, United States). After incubation in room temperature for 30 min the supernatant was removed as previously described in section “RRBS Library Preparation Using Ovation^®^ RRBS Methyl-Seq System (Ovation Method)” and beads were washed and re-suspended in 20 μl elution buffer (Qiagen, Germany). Adapter ligation was performed by adding 2 μl of NEXTflex^TM^ Bisulfite-Seq barcodes (Bio Scientific Corporation, United States) and ligase mixture to eluted DNA followed by overnight incubation at 16°C. Adapter-ligated DNA again was size selected by adding 1.5x of 20% PEG 8000/2.5 M NaCl (Amresco Inc., United States) followed by incubation at room temperature for 30 min. The product was placed on a side magnet; supernatant was removed, beads were washed two times in 70% EtOH and were re-suspended in 25 μl elution buffer (Qiagen, Germany). Prior to fragment amplification, different PCR amplification cycles (10, 13, 16, and 19 cycles) were tested. PCR products were run on a gradient 4–20% Criterion precast polyacrylamide TBE gel (Thermo Fisher Scientific, United States). Gradient gel further stained with SybrGold (Thermo Fisher Scientific, United States) and the efficiency of protocol were evaluated based on the appearing DNA bands. Later, size selected DNA fragments were bisulfite converted and product was cleaned up according to recommended protocol in the QIAGEN EpiTect kit (Gu et al., 2011). At the last step, converted DNA, PCR amplified using 13 amplification steps (PCR Primer 1: 5′- AATGATACGGCGACCGAGATCTACAC-3′, PCR Primer 2: 5′-CAAGCAGAAGACGGCATACGAGAT-3′) and PCR product (libraries) were further cleaned up using 1x SPRI beads as described earlier for the Ovation method.

### Illumina Sequencing

Eluted RRBS libraries from both protocols were quantified using the PicoGreen dsDNA absorbance method and were sent to Norwegian Sequencing Center. Sequencing was performed using Illumina HiSeq 2500 in the paired-end (2 × 150 bp) mode.

### Bioinformatics Analyses

#### Illumina Reads Quality Assessment and Trimming

The quality of paired-end Illumina reads first was assessed using fastQC software (v 0.11.8 for Linux). For reads obtained via the gel-free protocol, Illumina adapters and low-quality sequences (below 20 bp and Phred score of 30) were trimmed using Trim-galore software (v 0.4.4 for Linux) (Martin, 2011). For reads obtained via the Ovation protocol, manufacturer’s recommendations were followed for quality control and adapter trimming^1^. Then, additional nucleotides from the 5′ ends of adapter-removed reads, were further trimmed using a NuGEN-developed ‘trimRRBSdiversityAdaptCustomers.py’ script in Python (2.7.5 for Linux).

#### Mapping the Clean Reads With Reference Genome

Bull reference genome (*bosTau 9*) was downloaded from the UCSC database (UCSC, 2018) and was indexed using bismark_genome_preparation option in Bismark (v 0.19.0 for Linux) (Krueger and Andrews, 2011). After initial assessment of libraries (Table 2), only reads from the gel-free protocol were mapped to the reference genome. The mapping was carried out using Bismark and bowtie2 aligner (v 2.3.2 for Linux) (Krueger and Andrews, 2011) with following parameters [-n 0 −l 20 and –score-min (L, 0, −0.6)]. All covered Cs were used for calculation of global CpG methylation level in Bismark using following formula:% of global methylation = 100 ^∗^ number of methylated Cs/number of methylated Cs + number of unmethylated Cs.

#### Differential Methylation Analysis

In this study differentially methylated regions were identified between control (17 months old bulls) and test (14 months old bulls) groups. In brief, SAM-sorted alignment files from Bismark were analyzed using the methylKit package (v 1.6.1) (Akalin et al., 2012) in Rstudio (v 1.1.453 for Linux). First, reads containing CpGs with more than 99.9th percentile coverage were filtered out. Every single C was considered to calculate differentially methylated regions (DMRs). For this purpose, the genome was divided in 1000 bp tiles with sliding step 1000 bp, containing at least three mutually covered Cs in the CpG context. Average DNA methylation of each tile was calculated and in order to determine DMRs with *q-value* < 0.05 (filtered DMRs onward), logistic regression with a sliding linear model to correct for multiple comparisons was applied. In this study, hypermethylation and hypomethylation are defined as any positive and negative value for DMRs in the test group compared to the control group, respectively. Later, DMRs were categorized as those with < 10% differential methylation (DMRs_<__10__%_), those showing 10–25% (DMRs_10__–__25__%_) and those exhibiting > 25% methylation differences (DMRs_>__25__%_) and were used for downstream analyses.

#### Annotation of Differentially Methylated Regions

BED files containing gene and CpG annotation for the *bosTau9* assembly were downloaded from the UCSC table browser (UCSC, 2018). All DMRs with any level of hypo/hyper methylation were annotated with nearest (no specific cut off) transcriptional start site (TSS), genes elements (exons, introns, promoter, intergenic regions) and CpG features (CpG islands, CpG shore, other) using Genomation package (v 1.14.0) in Rstudio. Promoters and CpG shore were defined as ± 1000 bp and ± 2000 bp of the TSS and CpG islands, respectively.

#### Pathway Analysis

Corresponding GenBank accession IDs to annotated TSSs, were submitted to DAVID Bioinformatics resources for functional annotation (Huang da et al., 2009) for Gene Ontology (GO) analysis. Gene enrichment for each identified pathway was calculated using Fisher’s exact test and *p-value* was Benjamini adjusted for multiple testing and set to 0.05.

### Statistical Analyses

Statistical analyses were performed in Rstudio (v 1.1.383 for windows). In order to compare sperm quality parameters in fresh and frozen-thawed samples from 14 and 17 months old bulls, linear mixed models within the lme4 package were established using quality parameters of sperm cells and bulls age as response and independent variables, respectively. In addition, animals, semen concentration at the time of semen collection and pedigree information were included as random effects. The level of significance (*p-value*) was set to 0.05 except for DFI and HDS where, in order to minimize type I error, *p-values* were Bonferroni adjusted to 0.025. Results were plotted using GraphPad Prism (v 6.01 for Windows, GraphPad Software, San Diego, CA, United States). Venn diagrams were constructed using Venny online platform (Oliveros, 2015). Pathway analysis results were plotted using ggplot2 package (v 3.1.0) in Rstudio (Wickham, 2016).

## Results

### Sperm Quality Analyses in Young Norwegian Red Bulls

Sperm quality analyses results showed that some of the parameters were significantly different between the 14 and 17- months old bulls (Table 1). For instance, sperm concentration in 17 months old bulls was significantly higher compared to those 14 months old. Furthermore, both fresh and frozen-thawed samples from 14 months old bulls showed higher DFI compared to the 17 months old group. In addition, fresh sperm cells from 17 months old bulls showed significantly higher HDS (less condensed DNA) compared to those 14 months old. However, no significant changes in HDS between 14 and 17 months. of age were observed in frozen-thawed semen samples. The results further indicated that total sperm motility and progressivity in fresh semen from 17 months old bulls were significantly higher compared to 14 months old bulls. However, in frozen-thawed semen none of the sperm motility parameters was significantly different in bulls 14 months, compared to 17 months of age. Other sperm motion kinetic parameters showed no significance differences between 14 and 17 months old bulls (Supplementary Table 1).

**TABLE 1:** An overview of results (mean ± SEM) for different sperm quality parameters analyses for both fresh and frozen-thawed semen samples in 14 months (*n* = 9) and 17 months (*n* = 8) old Norwegian red bulls.

	Fresh semen		Frozen-thawed semen	
	14 months	17 months	14 months	17 months
Sperm concentration (10^6^ cells/ml)	974 ± 83.80	1330 ± 133.40*	NA	NA
DFI	2.78 ± 0.30	1.83 ± 0.20*	2.02 ± 0.13	1.64 ± 0.20*
HDS	0.67 ± 0.07	0.80 ± 0.10*	0.64 ± 0.05	0.88 ± 0.15
ATP (nM/10^6^ cells)	NA	NA	1.22 ± 0.13	1.16 ± 0.06
Viability (%)	NA	NA	62.15 ± 5.32	65.64 ± 2.85
Motility (%)	87.09 ± 1.51	98.10 ± 0.30*	58.45 ± 5.10	63.47 ± 2.95
Progressivity (%)	72.90 ± 2.00	87.23 ± 1.00*	52.16 ± 5.52	59.26 ± 3.16
Hyperactivity (%)	24.34 ± 1.83	24.21 ± 3.51	10.44 ± 2.24	12.50 ± 1.47

Asterisks indicate a significant difference between age in fresh or frozen-thawed samples based on linear mixed model. *p < 0.05 for all parameters except for DFI and HDS, where *p < 0.025 (Bonferroni corrected). NA, data not available; DFI, DNA fragmentation index; HDS, high DNA stainability; nM, nanomolar.

### Bioinformatics Analyses of RRBS Data

#### Comparison of RRBS Data Obtained Based on Ovation and Gel-Free Protocols

Table 2 compares the summary statistics for RRBS data obtained from two protocols. Surprisingly, no Illumina adapter contamination was detected for reverse reads in libraries constructed using Ovation method while both forward and reverse reads from RRBS libraries constructed based on gel-free method, showed Illumina adapter contamination. After quality control and trimming, 51% of reads were discarded in Ovation libraries, whereas quality control and trimming resulted in only 8% read loss in gel-free method. After trimming, reads with length < 50 bp and 100–150 bp were corresponding to 4 and 67% of all reads in libraries made according to gel-free protocol, respectively. Whereas reads with similar size in Ovation libraries were about 34 and 26% of total reads, respectively. This is particularly important, as longer reads tend to align better with reference genome in Bismark (Tran et al., 2014). Therefore, based on observed differences, we decided to conduct downstream analyses using RRBS libraries constructed based on the gel-free optimized method.

**TABLE 2:** An overview of summary statistic for RRBS libraries constructed based on Ovation RRBS Methyl-Seq and our previously optimized method (gel-free method).

RRBS protocol	Adapter contamination	Read loss after trimming	Reads length (bp) distribution after trimming
Ovation method	Detected only in forward reads	51%	< 50 34%
			50–99 40%
			100–150 26%
Gel-free method	Detected in both reads	8%	< 50 4%
			50–99 29%
			100–150 67%

**Paired-end reads from both methods were quality checked using fastQC software and trimming for Ovation libraries was performed according to manufacturer’s instructions. Read loss percentage was calculated as number of reads after trimming/number of reads before trimming. Bp, base pair.**

#### Basic Statistics of RRBS Libraries Constructed Based on Gel-Free Method

**FIGURE 1:**
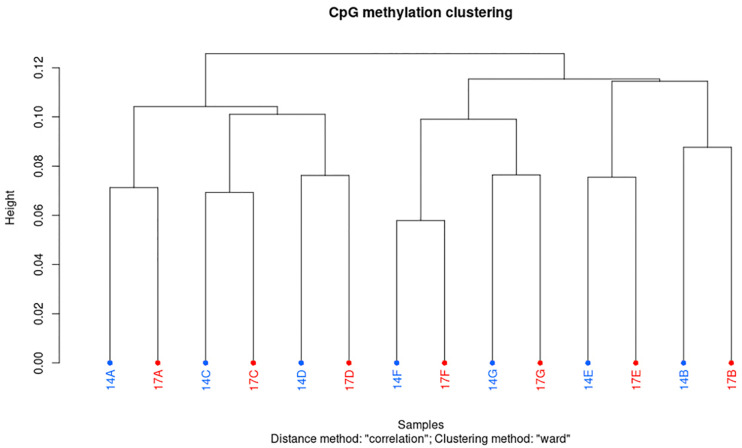
Hierarchical clustering of samples based on their CpG_*W1000*_ methylation levels in sperm DNA from Norwegian red bulls. Letters A to G prefixed by 14 or 17, indicating different bulls of age 14 and 17 months, respectively.

Using an in-house bioinformatics pipeline and after trimming the Illumina reads, 91% of reads were retrieved in libraries constructed based on the gel-free protocol. As shown in Table 3, this was equal to an average of 7.7 million reads per sample, 33.1% unique mapping efficiency, 15.9x read coverage and 99.1% conversion rate. Overall, minimum variation was observed between samples from different individuals and age regarding to retrieved clean reads, mapping efficiency, global CpG methylation, and bisulfite conversion rate (Table 3). Furthermore, CpG statistic revealed that generated libraries in average covered 4.4 million CpG, with methylation average of 40%. Mixed models indicated that none of mapping efficiency, global CpG methylation level and conversion rate parameters were significantly different (*p* < 0.05) in 14 months compared to 17 months old bulls. Cluster analysis based on methylation value of CpG_*W*__1000_ (i.e., CpGs that have fallen into a 1000 bp tiles across the genome) in each sample, showed that samples are distributed in two main clusters. However, within the main clusters, samples from the same individuals but different age always sub-clustered together (Figure 1). Furthermore, Pearson’s correlation coefficient based on methylation value of CpG_*W*__1000_ indicated a very high positive correlation between samples in term of global methylation profile (Pearson’s correlation coefficient ≥ 0.95) (Supplementary Table 2).

**TABLE 3:** An overview of basic statistic for RRBS libraries constructed based on the gel-free protocol.

Sample ID	Total reads	Clean reads after trimming	Read coverage (X)	Unique mapping efficiency (%)	Global CpG methylation (%)	Number of covered CpGs	Bisulfite conversion rate (%)
14 A	8,092,814	7,424,375	15.3	32.3	40.4	4,287,779	99.2
17 A	8,769,052	8,037,908	16.2	29.2	38.4	4,161,727	99.2
14 B	8,187,841	7,514,351	15.4	30.5	38.0	3,867,873	99.2
17 B	7,916,740	7,242,150	15.0	30.2	37.6	3,941,237	99.2
14 C	9,235,837	8,452,463	17.4	33.3	40.3	4,792,957	99.2
17 C	8,208,151	7,515,657	15.3	33.0	39.9	4,267,681	99.2
14 D	8,608,751	7,932,141	15.6	33.3	39.6	4,529,706	99.3
17 D	6,947,434	6,395,163	12.4	32.6	39.6	3,918,954	99.2
14 E	7,745,029	7,098,249	14.6	33.9	40.2	4,230,165	99.2
17 E	8,179,041	7,538,949	15.7	34.4	40.7	4,384,431	99.2
14 F	9,561,827	8,631,458	18.2	33.9	41.2	4,855,729	99.0
17 F	11,204,211	10,288,338	21.6	34.8	41.4	5,383,672	98.9
14 G	6,739,955	6,197,336	13.1	35.2	40.7	3,947,850	98.8
17 G	8,406,205	7,643,950	16.4	37.0	42.7	4,819,724	98.7

**Libraries were constructed from sperm DNA collected from the same young Norwegian red bulls (n = 7) at two different ages. Letters A to G prefixed by 14 or 17, indicating different bulls of age 14 and 17 months, respectively. Clean reads were obtained after adapter and low-quality trimming of Illumina sequencing reads (total reads). Read coverage was calculated by number of bp in the clean reads/number of bp at in silico MspI-digested bosTau9 genome. Mapping efficiency shows the percentage of uniquely mapped clean reads with the reference genome. CpG methylation shows the percentage of global methylation in clean reads. Downstream analyses were performed based on all covered CpGs. Bisulfite conversion rate shows the proportion of Cs, which converted to uracil during bisulfite conversion process.**

#### Differential Methylation Analysis

**FIGURE 2:**
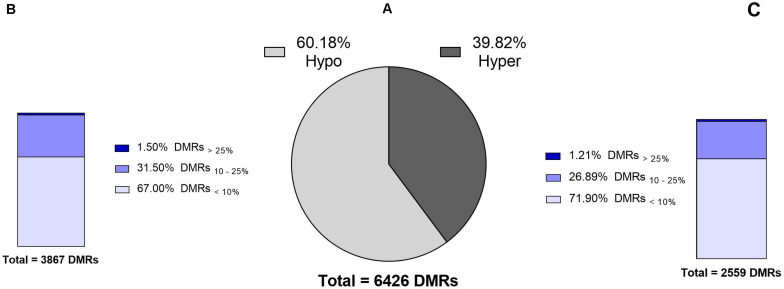
Numbers and levels of significant DMRs identified in the sperm DNA methylome between identical 14 months old bulls (test) and 17 months old bulls (control). **(A)** Total number of significant DMRs indicating that 60% are hypomethylated in the test group compared to the control group. **(B,C)** Different levels of hypo- and hypermethylated regions in the test group compared to the control group. Explanation of percentage ranges: DMRs_<__10%_ indicating regions with less than 10% difference in DNA methylation, DMRs_10__–__25%_ indicating regions having between 10 and 25% difference in DNA methylation, DMRs_>__25%_ indicating regions having over 25% difference in DNA methylation.

Differential methylation analysis were performed using a tile-based approach. This resulted in identification of 131,073 DMRs between test (14 months old) and control (17 months old) bulls. However, after setting the level of significance to *q-value* < 0.05, a total number of 6426 DMRs (filtered DMRs) were detected with varying levels of methylation ranging from 0 to 38%. Majority of filtered DMRs (60%) were found to be hypomethylated in the 14 months old group relative to the control group (Figure 2A). Distribution of DMRs exhibiting varying degrees of methylation differences in hypomethylation and hypermethylation groups were similar; ∼70% of DMRs showed less than a 10% difference in methylation, and ca. 1.5% of DMRs had over a 25% difference in methylation levels (Figures 2B,C).

#### Annotation of Differentially Methylated Regions With Gene and CpG Features

**FIGURE 3:**
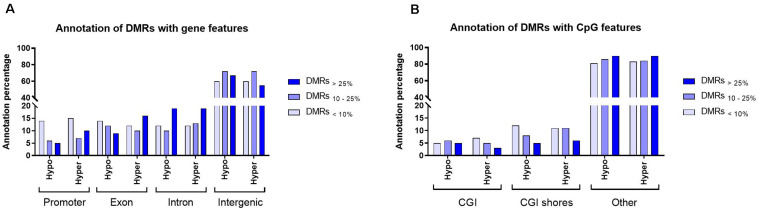
An overview of the distribution of filtered DMRs in the *bosTau9* genome. **(A)** Annotation of the hypo- and hypermethylated regions with gene features. **(B)** Annotation of the hypo- and hypermethylated regions with CpG features. Hypo, hypomethylated regions; Hyper, hypermethylated regions; CGI, CpG island. Explanation of percentage ranges: DMRs_<__10%_ indicating regions with less than 10% difference in DNA methylation, DMRs_10__–__25%_ indicating regions having between 10 and 25% difference in DNA methylation, DMRs_>__25%_ indicating regions having over 25% difference in DNA methylation.

In this study, all filtered DMRs with any level of methylation differences (DMRs_<__10__%_, DMRs_10__–__25__%_ and DMRs_>__25__%_), were considered for downstream analyses. The filtered DMRs were annotated with gene and CpG features. The analyses showed that, on average, 65% of the filtered DMRs were present in the intergenic regions. Annotation of DMRs in both hypomethylation and hypermethylation groups within promoters and introns showed similar trend. For instance, DMRs_<__10__%_ and DMRs_>__25__%_ were the major groups that annotated within promoter and intron regions, respectively (Figure 3A). For CpG features, on average, over 85% of filtered DMRs in both hypomethylation and hypermethylation groups were annotated within regions outside of CpG islands (CGI)/CpG shores. A majority of annotated DMRs within these external regions exhibited methylation differences exceeding 25%. Only around 15% of filtered DMRs were annotated within CGI/CpG shores and most showed less than a 10% methylation difference (Figure 3B).

**FIGURE 4:**
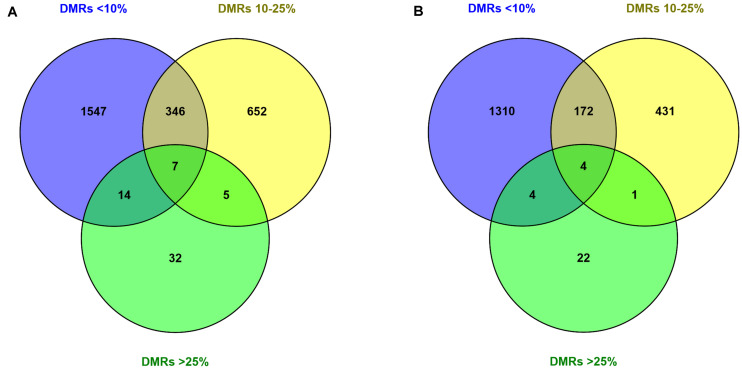
Annotation of the filtered DMRs with varying degrees of methylation difference to the nearest TSSs. Numbers inside the circles, showing the number of closest unique TSSs (duplicated TSSs removed) to DMRs in **(A)** hypomethylation and **(B)** hypermethylation groups. Explanation of percentage ranges: DMRs_<__10%_ indicating regions with less than 10% difference in DNA methylation, DMRs_10__–__25%_ indicating regions having between 10 and 25% difference in DNA methylation, DMRs_>__25%_ indicating regions having over 25% difference in DNA methylation.

Next, the nearest transcription start sites (TSSs) to filtered DMRs were extracted (Figures 4A,B). This resulted in a greater number of TSSs in the hypomethylation group (2982 TSSs) compared to the hypermethylation group (2129 TSSs). However, in both hypomethylation and hypermethylation groups, a majority of TSSs were associated with DMRs_<__10__%_, followed by DMRs_10__–__25__%_ and DMRs_>__25__%_. Furthermore, 474 TSSs associated to DMRs_<__10__%_ were annotated with both hypo- and hypermethylated regions. This number for the DMRs_10__–__25__%_ was 156 TSSs and no commonly annotated TSS was found between hypo- and hypermethylated regions in the DMRs_>__25__%_ group (Supplementary Figure S1 and Table 3).

#### Pathway Analysis

**FIGURE 5:**
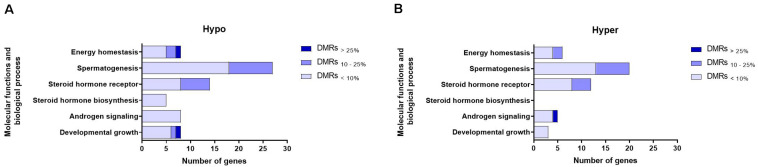
Numbers of genes representing different molecular functions and biological processes related to sexual maturation in **(A)** hypomethylated (Hypo) and **(B)** hypermethylated (Hyper) groups and displayed according to their association with DMR-groups exhibiting varying degrees of methylation differences. Explanation of percentage ranges: DMRs_<__10%_ indicating regions with less than 10% difference in DNA methylation, DMRs_10__–__25%_ indicating regions having between 10 and 25% difference in DNA methylation, DMRs_>__25%_ indicating regions having over 25% difference in DNA methylation.

Genes whose TSSs were annotated with DMRs were first identified and subsequently subjected to pathway analysis. We were particularly interested in genes associated with biological processes and molecular functions related to sexual maturity such as androgen signaling, steroid hormone biosynthesis, steroid hormone receptor signaling, spermatogenesis and developmental growth. Figures 5A,B show that the numbers of such genes were higher in the hypomethylation group compared to the hypermethylation group. Steroid hormone biosynthesis, identified exclusively in hypermethylation group. Moreover, genes whose TSSs were annotated with the DMRs_<__10__%_ and DMRs_>__25__%_ groups represented those functions to the greatest and least extent, respectively. In both hypo- and hypermethylation groups, functions including spermatogenesis, followed by steroid hormone receptor and energy homeostasis, were represented by the highest numbers of genes (Supplementary Table 4).

**FIGURE 6:**
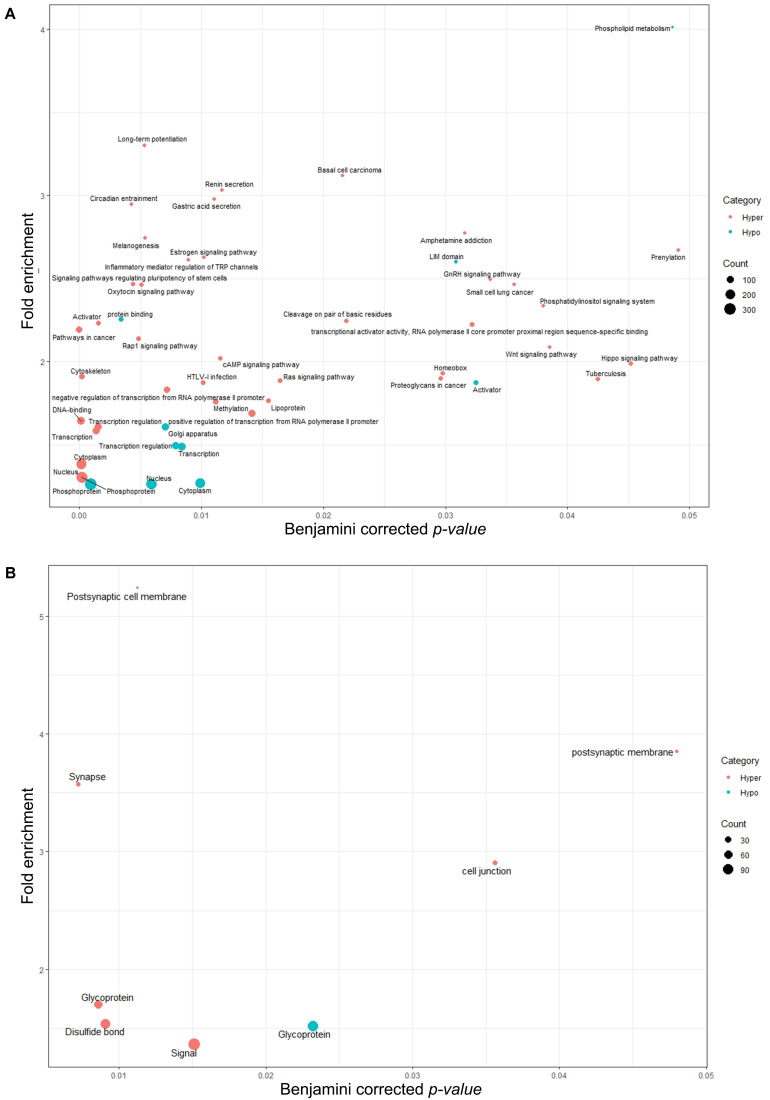
Pathway analysis for annotated genes associated with **(A)** filtered DMRs with less than a 10% methylation difference and **(B)** filtered DMRs with a 10–25% methylation difference. No significant pathways were identified for annotated genes associated with DMRs over 25% methylation difference. GO terms are plotted in function of their Benjamini corrected *p*-value (*x*-axis) and fold enrichment (*y*-axis). Gene count size key shows the number of genes involved in that particular pathway. Hypo, hypomethylated regions (referring to TSSs annotated with hypomethylated regions in test group); Hyper, hypermethylated regions (referring to TSSs annotated to hypermethylated regions in test group).

As shown in Figure 6, similar to biological processes and molecular functions, the majority of identified pathways (49 pathways) were in association with genes annotated with DMRs_<__10__%_. Only eight pathways were linked to genes annotated with DMRs_10__–__25__%_. None of the identified pathways exhibited significant association with DMRs_>__25__%_. Some of the hormonal pathways (gonadotropin-releasing hormone, estrogen and oxytocin signaling) and sperm function related pathways (disulfide bond and glycoprotein) were exclusively identified in the hypermethylation group of test samples (14 months old bulls). In other words, genes associated with those pathways were annotated with hypomethylated regions in more mature, 17 months old, bulls. Although the number of annotated TSSs to DMRs (with any level) was higher in hypomethylation groups compared to the hypermethylation groups (Figure 4), the number of pathways represented by genes harboring those TSSs showed an opposite trend (Figure 6). However, the majority of identified pathways in the DMRs_<__10__%_ hypomethylation group exhibited stronger *p-values* (Figure 6A).

## Discussion

In this study, sperm quality was assessed in 14 and 17 months old NR bulls. Furthermore, DNA methylation patterns were elucidated in sperm cells from the same individuals using RRBS data generated from comparative library construction where two protocols were tested.

Our results showed that the number of sperm cells in ejaculates from NR bulls significantly increased with aging. These results support previous findings where total sperm count was higher in post-pubertal Holstein bulls compared to 4 months younger bulls (Devkota et al., 2008; Wu et al., 2020). Sperm DFI results (Table 1) indicate that DNA integrity also improves with aging. This can be explained by ongoing sperm nucleus development from 14 to 17 months of age as it has been reported that sperm DNA is more compact in older bulls rendering it less prone to fragmentation (Andrabi, 2007). In addition, sperm DFI was reduced in frozen-thawed semen samples compared to fresh samples in both age groups. It seems possible that sperm cells with higher DFI in fresh semen may not tolerate the cryopreservation process and became degraded during cryopreservation and thawing, hence not falling into the gate defined as sperm cells based on electronic volume in the flowcytometry analysis. However, the observed differences are small and less likely to be of biological importance. The HDS results presented here indicate that the degree of sperm chromatin compactness was reduced in fresh NR ejaculates at 17 compared to 14 months of age. This must, however, be further investigated using a larger number of samples. Furthermore, both total sperm motility and sperm progressivity were significantly increased in fresh semen from bulls of age 17 compared to 14 months. This trend was also observed for frozen-thawed samples, although the changes were not significant. These results are in agreement with observations from previous research where sperm cell motility in fresh samples were positively correlated with increasing age in Holstein bulls (Devkota et al., 2008; Murphy et al., 2018) and Nelore bulls (Reis et al., 2016). Overall, the results from sperm quality analyses show that sperm cells from NR bulls at 17 months of age displayed higher sperm quality compared to 14 months of age. Although sexual maturation lasting up to 50 weeks of age (Rawlings et al., 2008), previous evidences have documented that bull sperm quality increased even after puberty (Brito et al., 2004). Therefore, these results are likely to be related to the well-known sexual maturation process.

Read quality control is an important initial step in next generation sequencing data processing. The Trim-galore software has been recommended for trimming the low-quality reads in RRBS libraries (Wreczycka et al., 2017). Although RRBS libraries from the Ovation method were constructed, evaluated and trimmed according to criteria recommended by the manufacturer, surprisingly we did not detect any Illumina adapter sequences in the reverse reads of libraries. This observation might partially be explained by the sequencing technique and source of DNA. Although according to manufacture recommendation, Ovation method is compatible with paired-end sequencing, previous results employing this method, sequenced the libraries in a single-end mode, and not paired-end, mode (Pilsner et al., 2018; Chen et al., 2019; Paul et al., 2019; Rashid et al., 2020). The same studies applied this method to study DNA methylome in rat, mice and humans brain/sperm cells while, according to manufacturer’s recommendation, the Ovation method is designed to generate RRBS libraries from human genomic DNA (NuGEN, 2020). Length distribution after trimming also revealed that libraries constructed using the Ovation method had several peaks reflecting different fragment sizes specifically of short length, whereas reads from the gel-free method revealed only one major peak of fragments greater than 130 base pairs long. This is an important factor as it has been shown that longer reads align better and more specifically to a reference genome (Tran et al., 2014). To our knowledge, this was the first time the Ovation^®^ RRBS Methyl-Seq system has been applied to study DNA methylation in bull sperm. However, further work is required to successfully adopt Ovation system for studying the bull sperm DNA methylome.

Basic statistics of sequencing results from the RRBS libraries constructed using the gel-free protocol indicated very consistent and reproducible bisulfite conversion between samples. The average conversion rate of 99.1% is higher and equal to previously published whole genome bisulfite sequencing (WGBS) (Duan et al., 2019) and RRBS results (Jiang et al., 2018) for bull sperm cells, respectively. Although in this study, the *bosTau9* genome was used as the reference genome and some relaxed alignment parameters were applied, the average mapping efficiency of 33.1% was much higher than previously reported results for RRBS libraries in bull sperm cells (Jiang et al., 2018).

Results further indicated a 40% global DNA methylation level in NR bull sperm cells. Previous studies showed that, in general, global DNA methylation level is low in bull sperm cells. For instance, using a luminometric methylation assay an average of 45% (Perrier et al., 2018) and using RRBS, an average of 35% (Jiang et al., 2018) global CpG methylation in bull sperm cells has been reported. Similarly, low global CpG methylation was also reported for ten different cattle tissues using RRBS (Zhou et al., 2016). Previously, we reported an average of 33% of global methylation in boar sperm cells using a gel-free RRBS technique (Khezri et al., 2019). However, it has been shown that global sperm DNA methylation in bulls can reach 75% as documented using WGBS (Zhou et al., 2018). One should keep in mind that the applied RRBS method in this study focuses on a small subset (CpG island) of the compact sperm genome where methylation levels are generally low (Suzuki and Bird, 2008). In addition, differences in global bull sperm DNA methylation described in the literature might be explained by different laboratory techniques, instrumental platforms, bioinformatics workflows, reference genome versions utilized for read alignment and interspecies differences in sperm DNA methylation patterns.

Here, no significant associations between sperm global CpG methylation and age were found. These findings are further supported by Pearson correlation and cluster analyses, where a high positive correlation between samples from both age groups was observed. In addition, samples from both age groups of the same individuals always clustered together, which suggests that, in this study, individual effects on global sperm DNA methylation are probably more pronounced than age effects. Considering uniform condition and environment for raising and feeding the bulls, it is least likely that individual differences in DNA methylation here is driven by environmental factors. However, in addition to environmental factors, it has been shown that individual differences in sperm DNA methylation may be explained by epigenetic polymorphism phenomenon and interindividual genetic diversity (Kiefer and Perrier, 2019). In agreement with global sperm DNA methylome results presented here, previous research reported that DNA methylation levels in bull sperm is dynamic during puberty, becoming stable after the age of 1 year (Lambert et al., 2018). In parallel with global methylation analysis, differential methylation analysis, showed an increasing trend of DNA methylation in the control group (sperm DNA from 17 months old bulls) compared to test group (sperm DNA from 14 months old bulls). Although, 70% of identified differentially methylated regions, displayed less than 10% methylation difference, we believe that this further highlights the possibility of an existing relationship between differentially methylated regions and sexual maturation in NR bulls. This hypothesis is supported by previous studies in Holstein bulls where more methylated regions were found in sperm cells from 16 months bulls compared to 10 months bulls (Lambert et al., 2018). Similar findings were reported in one Japanese black bull (at 14, 19, 28, 54, and 162 months of age), where authors identified eight CpGs that exhibited an age-dependent increase in their methylation levels (Takeda et al., 2019).

The distribution of DMRs demonstrated here showed that the majority lay within intergenic regions and regions outside CpG Islands/CpG shores. Similar trends have been reported in boar (Hwang et al., 2017; Khezri et al., 2019) and bull (Jiang et al., 2018; Perrier et al., 2018) sperm DNA. Previous research has shown that CpG Islands and CpG shores, in parallel with promoters, play an important role in regulation of transcription (Deaton and Bird, 2011; Long et al., 2017). Although only a small percentage of DMRs were annotated with CpG Island/CpG shores and promoters here, the majority of annotated DMRs exhibited less than a 10% methylation difference. This further suggests similar DNA methylation profiles in these regions in sperm samples from NR bulls at age 14 and 17 months.

GO analysis results for the DMR_>__25__%_ group further showed that molecular functions/biological processes such as energy homeostasis, developmental growth and androgen signaling could be driven by Cytochrome B5 Reductase 4 (*CYB5R4*), Phospholipase C Beta 1 (*PLCB1*) and NK3 homeobox 1 (*NKX3-1*) genes, respectively. However, these genes are not specific for reproduction or sexual maturation. For instance, previous research demonstrated that the *CYB5R4* gene could be consider as one of the candidate gene for quantitative trait locus studies for the oleic acid percentage in Japanese Black cattle (Kawaguchi et al., 2019). In other research, the *PLCB1* gene was identified in oxidative stress response and heat tolerance in Dehong humped cattle (Li et al., 2020). Furthermore, the transcription factor *NKX3-1* was proposed as a possible regulator of gene expression in the endometrium of cattle who received n-3 polyunsaturated fatty acid as a feed supplement (Waters et al., 2014). In addition, steroid hormone biosynthesis was the only biological process that was exclusively identified in the DMR_<__10__%_ hypomethylation group. Several genes also identified, such as cytochrome P450 superfamily members (*CYP1B1, CYP11A1, and CYP2E1*), steroid 5 alpha-reductase 2 (*SRD5A2*) and steroid sulfatase (*STS*) are annotated to be involve in steroid hormone biosynthesis. Given their annotated molecular functions/associated biological processes, genes identified in this study may contribute to age-dependent reproductive capacity in NR bulls.

Our analyses did not show any significant pathways connected to genes annotated with DMR_>__25__%_. These findings are in line with previous research from Holstein bulls, where no significant DMR-associated pathways were found in sperm samples collected at 12 and 16 months of age (Lambert et al., 2018). For the DMR_10__–__25__%_ group, a total number of eight significant pathways including sperm-relevant pathways such as “disulfide bond” and “glycoprotein” in 14 months old NR bulls were identified. “Disulfide bond” was exclusively identified in the hypermethylation group. It has been shown that disulfide bonds are essential for protamine function and DNA packaging in bull sperm chromatin (Hutchison et al., 2017). Although the number of bulls was limited here, fresh semen samples from 14 months old NR bulls exhibited higher degree of chromatin compaction compared to 17 months old bulls (Table 1). These results suggest a possible link between sperm DNA hypermethylation and DNA packaging via protamine function. Similar possible contribution of DNA methylation to nucleosome rigidity via histone function, has previously been suggested in human somatic cells (Choy et al., 2010; Lee and Lee, 2012). Furthermore, the pathway “glycoprotein” was identified in both hypo and hyper DMR_10__–__25__%_ with a stronger *p-values* in the hypermethylation group. “Glycoproteins” have been identified in the sperm plasma membrane and play an important role in mammalian fertilization (Tecle and Gagneux, 2015). Further research is required to shed light on compositions of sperm glycoproteins during bull sexual maturation. The highest numbers of identified pathways with significant *p-values* were found to be related to genes annotated with DMR_<__10__%._ In the study conducted by Lambert et al. (2018), identified DMRs in sperm cells from bulls at 10 and 16 months of age were associated with pathways related to sperm function, including androgen hormone signaling. Here, we identified other hormonal pathways such as GnRH, estrogen and oxytocin signaling pathways, which were exclusively related to DMR_<__10__%_. This further emphasizes the importance of hormonal signaling in development and sexual maturation. However, pathway analysis results need to be interpreted with caution for two main reasons. First, it has been recommended to avoid using differential DNA methylation level cut off percentages less than 5% in DMR-analysis due to the minimal effects on gene expression they exercise (Wreczycka et al., 2017). Second, a moderate number of genes annotated with DMRs overlapped between hypo- and hypermethylation groups. How transcriptional regulation can be exerted via TSSs proximal to both hypo- and hypermethylated regions is not clear, especially in sperm cells that are relatively transcriptionally silent. Therefore, further research using transcriptome analysis of *in vitro* produced embryos, fertilized with sperm cells from wider age groups of young NR bulls is recommended.

## Conclusion

The purpose of the present research was to study the sperm DNA methylome, in parallel with sperm quality assessment, in similar NR bulls both at 14 and 17 months of age. Although the number of tested bulls were limited, the present study found that with increasing age of young bulls, sperm quality increased. Furthermore, a gel-free, multiplexed method to construct RRBS libraries from frozen-thawed bull sperm cells was found to be reproducible. The current results showed that sperm DNA methylation in 14- and 17-months-old NR bulls was similar globally, while marginally different regionally. Taken all together, identified DMRs even with low levels of methylation differences, in parallel with sperm quality results, offers some useful insight into the reproductive capacity of genomic selected young NR bulls.

## Data Availability Statement

The datasets presented in this study can be found in online repositories. The names of the repository/repositories and accession number(s) can be found below: https://www.ebi.ac.uk/ena, PRJEB37763.

## Ethics Statement

Ethical review and approval was not required for the animal study because sperm cells that we used in this research routinely collected from bulls owned by breeding company Geno in Norway. However, the bulls were housed and cared for according to international guidelines and regulations for keeping bulls in Norway, at Geno artificial insemination (AI) station, in Hamar, Norway.

## Author Contributions

AK performed the sperm motility assay, bioinformatics and biostatistics analyses with inputs from RA as well as RW and wrote the manuscript. BN, E-BS, and TZ performed and drafted the viability assay, sperm chromatin integrity analysis and ATP content assay, respectively. E-BS prepared RRBS libraries with inputs from RW. FM and EK did conceptualization and original project draft. All authors were involved in the planning of the experiments and provided useful inputs, interpreted the data, read, edited, and approved the manuscript.

## Conflict of Interest

The authors declare that the research was conducted in the absence of any commercial or financial relationships that could be construed as a potential conflict of interest.

## Footnotes

1 https://github.com/nugentechnologies/NuMetRRBS
