# Supplementary material for: Sperm DNA Hypomethylation Proximal to Reproduction Pathway Genes in Maturing Elite Norwegian Red Bulls
Source: Front Genet. 2020 Aug 11;11:922. doi: 10.3389/fgene.2020.00922 (PMC7431628; doi:10.3389/fgene.2020.00922)
Supplement: Supplementary file 1 [file Data_Sheet_1.DOCX]

Supplementary figure and table legends

**Table S1.** An overview of sperm motion kinetics results (mean ± SEM) obtained from CASA for both fresh and frozen-thawed semen samples in 14-mo. (n = 9) and 17-mo. (n = 8) old Norwegian red bulls. Curvilinear velocity (VCL, µm/s), straight-line velocity (VSL, µm/s), average path velocity (VAP, µm/s), straightness of the average path [STR (%) = VSL/VAP], linearity of the curvilinear path [LIN (%) = VSL/VCL], Wobble [WOB (%) = VAP/VCL], lateral displacement of sperm head (ALH, µm) and beat cross frequency (BCF, Hz).

**Table S2.** Correlation analysis based on methylation value of CpG_W1000_ (i.e., CpGs that have fallen into a 1000 bp tiles across the genome) in each sample. Numbers in each cell represent the pairwise Pearson’s correlation scores. Letters A to G prefixed by 14 or 17, indicating different bulls of age 14 mos. and 17 mos., respectively.

**Table S3.** List of closest transcription start sites (TSSs) to differentially methylated regions (DMRs). All DMRs with less than 10% (<10%), between 10 -25% (10-25%) and over 25% (25%<) methylation differences in both hypomethylating (hypo) and hypermethylation (hyper) were annotated with closest TSSs. Distance to feature; distance between DMRs and closest TSSs. Feature name; corresponding GenBank accession IDs to annotated TSSs that were applied for pathway analyses. Feature strand: DNA strand that TSSs were observed.

**Table S4.** List of genes (corresponding GenBank accession IDs to annotated TSSs) involved in different biological process and molecular functions relevant to sexual maturity in NR bulls. All DMRs with less than 10% (<10%), between 10 -25% (10-25%) and over 25% (25%<) methylation difference in both hypomethylating (hypo) and hypermethylation (hyper) were annotated with closest TSSs.

**Figure S1.** An overview of mutually annotated TSSs to both hypometylated (hypo) and hypermethylated (hyper) regions with A) less than 10% (<10%), B) between 10 -25% (10-25%) and C) over 25% (25%<) methylation differences.
