# Supplementary figures and images for: Sperm DNA Hypomethylation Proximal to Reproduction Pathway Genes in Maturing Elite Norwegian Red Bulls
Source: Front Genet. 2020 Aug 11;11:922. doi: 10.3389/fgene.2020.00922 (PMC7431628; doi:10.3389/fgene.2020.00922)

## Slide 1
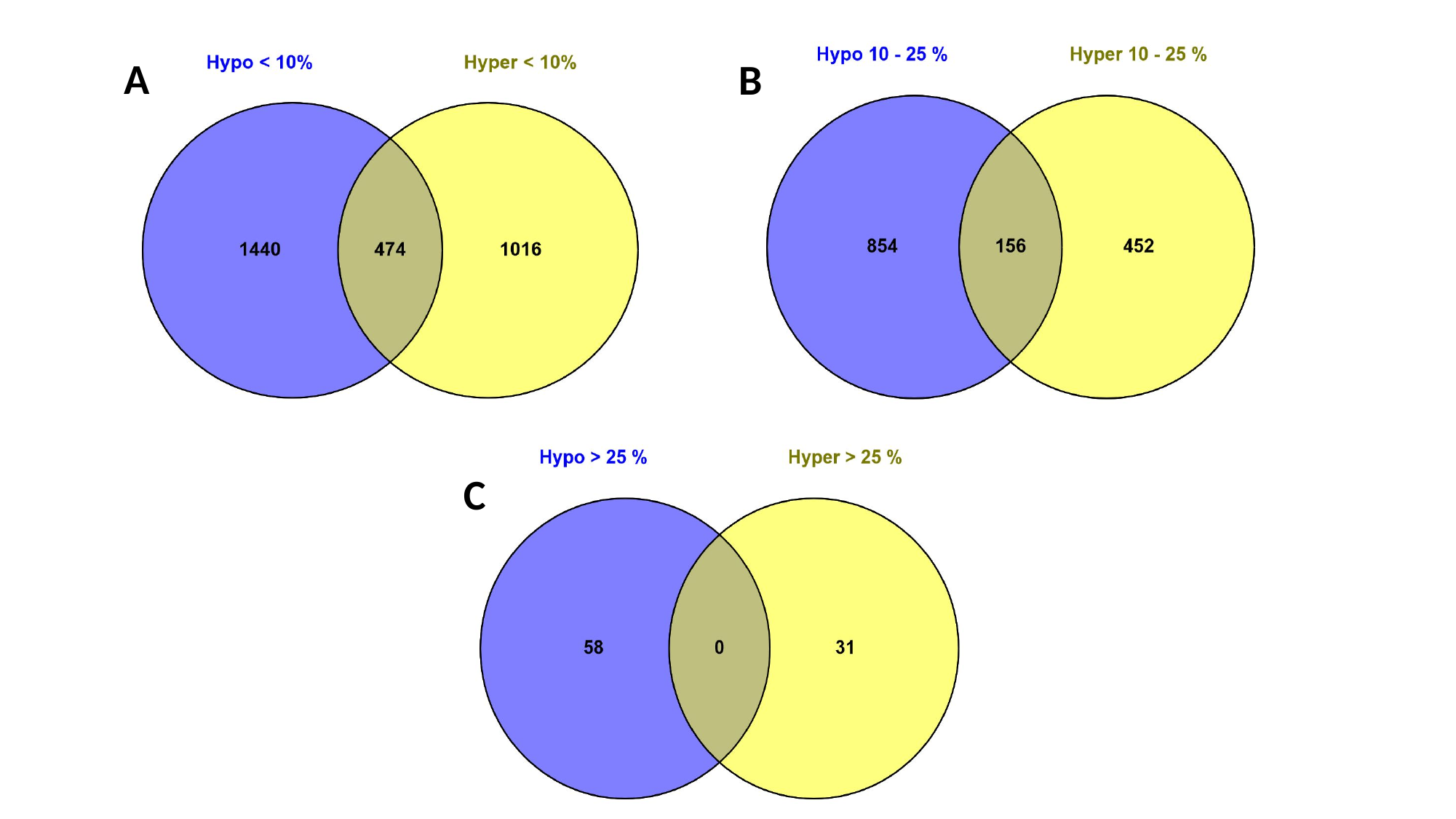

A
B
C

Supplement: Supplementary file 2 [file Presentation_1.PPTX]
